# Research evidence from studies on filial imprinting, attachment, and early life stress: a new route for scientific integration

**DOI:** 10.1007/s10211-020-00346-7

**Published:** 2020-06-08

**Authors:** Erwin Lemche

**Affiliations:** Section of Cognitive Neuropsychiatry and Centre for Neuroimaging Sciences, Institute of Psychiatry, Psychology & Neuroscience, King’s College London, De Crespigny Park, London, SE5 8AF UK

**Keywords:** Ethology, Filial imprinting, Pet attachment, Attachment classification, Mental representation, Bonding, Early life stress, Oxytocin, Triiodothyronine, Type 2 iodothyronine deiodinase, CD38, mTORC1, c-Fos, GABA, Amygdala, IMM, MNM

## Abstract

Attachment is a concept that was developed and researched in developmental psychology in uptake of findings on filial imprinting from ethology. In the present period, however, attachment concepts are increasingly applied to and investigated in animal research, thereby translating back criteria that were established for human infants. It herein appears that findings on filial imprinting are becoming more and more forgotten, whilst basic findings in human infants are not reflected in investigations on attachment in animals. To re-integrate both domains, the present article undertakes the effort in briefly reviewing and recapitulating basic findings in human attachment and recent research on filial imprinting. In specific, replicated were critical roles of the conversion of thyroid prohormone by 2 iodothyronine deiodinase (Dio2) into triiodothyronine (T_3_) in the regulation of the timing of imprinting learning. Because of the interactions of T_3_ with oxytocinergic and dopaminergic neurones of the hypothalamic paraventricular nucleus, these findings provide new neuroendocrinological insight for possible relations with both attachment and metabolic sequelae of early life stress. Necessary is a mutual integration of all recent advances in the yet separated fields.

## Introduction: imprinting vs. attachment

The accumulation of new research findings on attachment patterns in research in companion mammals (Vitale et al. 2019), specifically with human owners and infant canine and feline pets, are puzzling because of contradicting expectations. Hence, there is a requirement for closer inspection of such results and for clarification of concepts of ethological filial imprinting, acquisition of infant-parent bonds and human attachment theory. Whilst primates are documented to produce behaviours resembling human attachment patterns (Bauman et al. 2004; Miller et al. 1990), other studies on non-human mammals, such as *Octodon degus* (Zehle et al. 2007) or domesticated sheep (Kendrick et al. 1998; Neumann 2008), did not conclude in respective patterns, despite exclusive familial bonds existing in these species.

## Origins of filial imprinting

Human ethology claimed that mechanisms similar to imprinting in higher vertebrates are underlying human attachment development. In this respect, relevant sensitive periods or phases (Immelmann and Suomi 1982) with key stimulation were assumed also for human infants. Filial imprinting was first reported by Douglas Spalding (1841–1877) (Spalding 1872), and later by Oskar Heinroth (1871–1945) and Konrad Lorenz (1903–1989). Cues leading to imprinting memories were systematically examined in experimentation by Gabriel Horn (1927–2012), isolating the avian structure intermediate medial hyperstriatum ventrale (IMHV) (2004 renamed into IMM, see the “Brain bases of imprinting” section below) as substrate of their cerebral engrammes. That imprinting effects in mammals are formative for later mating preferences was concluded from goat-sheep cross-fostering (Kendrick et al. 1998). Harry Harlow’s deprivation studies demonstrated imprinting preferences of infant macaques for cloth/wire-surrogate mothers even in absence of any caretaking efforts (Harlow 1958).

## Brain bases of imprinting

It is nowadays accepted that filial imprinting is a rapid learning process deriving from innate priors or preferences, and as a consequence of this learning, not only the imprinted mother is henceforth recognised, but also other conspecifics, siblings in particular (Versace et al. 2018). The critical brain substrates known in chicks (*Gallus gallus domesticus*) are combined basal ganglia and neocortical association areas (homologous to V5 in humans) of the telencephalon, the intermediate medial mesopallium (IMM) that has a central role in imprinting via visual cues (Horn 2004), and the medio-rostral nidopallium/mesopallium (MNM) that was found the imprinting-relevant region for auditory cues (Braun et al. 1996; Maier and Scheich 1983).

Later, two arcopallial amygdaloid structures have also been identified in avian species (Yamamoto et al. 2005): Nucleus taeniae was found comparable with mammalian medial amygdala and avian subpallial amygdala resembles the extended amygdala in mammals. Electrophysiological experimentation utilising the expression of transcription factor c-Fos as indirect indicator of neuronal activity in domestic chicks revealed that these nuclei respond selectively to hens rather than scrambled pictures (Mayer et al. 2019). These findings suggest a critical role for the amygdaloid nucleus taeniae in innate preferences and in filial imprinting, and further support human findings of the amygdala as subserving attachment regulation (Lemche et al. 2005) (the “Cerebral regulation of attachment security” section below).

## Molecular mechanisms of imprinting memory consolidation

Molecular and plasticity mechanisms are required for long-term storage of imprinting memories (Brown and Banks 2015) in the left and right IMM, respectively: the left IMM is more involved in synaptic plasticity for preference retention, whereas the right IMM is critical for lasting memory consolidation (Brown and Banks 2015). Sleep after imprinting is further essentially required for successful long-term storage.

A timeline of molecular signatures related to electrophysiological changes following imprinting training (reviewed in Horn (2004); Solomonia and McCabe (2015)) related to synaptic stabilisation has been described. Initially, learning-related increase in Fos expression in the left IMM was restricted to neurones immunopositive for GABA and the gliotransmitter taurine, leading into enlargement of pools of GABA, taurine and glutamate, with subsequent increases in NMDA receptor numbers. After 6 h, post-synaptic densities (PSDs) in the IMM increase, accompanied by increased expression of neuronal cell adhesion molecules (NCAM), where NCAMs 120 and 180 in the left IMM at 25 h possibly reflect onset of myelination cycles (for review see Lemche (1997)) indicating connectivity stabilisation (Pan et al. 2020). The endoplasmic reticulum-derived cell adhesion molecule protein cognin, which binds thyroid hormones, may then trigger further related neuronal differentiation pathways (Solomonia and McCabe 2015).

## Sensitive period and endocrine/subcellular signalling

Pharmacological experimentation combined with PCR and immunoblotting in domestic chicks (*Gallus gallus domesticus)* indicated that thyroid hormone 3,5,30-triiodothyronine (T_3_) determinates onset and timing of the sensitive period, that activation of neuronal metabotropic GABA_B_ receptors in the IMM triggers imprinting projections, and that ionotropic GABA_A_ receptor activation terminates respective dendritic outgrowth towards arcopallium and intermediate hyperpallium apicale (IMHA) (Aoki et al. 2018; Aoki et al. 2015; Yamaguchi et al. 2012). Thyroid prohormone T_4_ is converted by type 2 iodothyronine deiodinase (Dio2) into T_3_, and both thyroid hormones modulate the onset of the sensitive period, through activation of neuronal thyroid receptors (TR), but without apparent involvement of genomic mechanisms (Yamaguchi et al. 2012).

T_3_ is thus determinative for both sensitive period and as a memory primer for extended memory content (Yamaguchi et al. 2012), through activating TRs, specifically for biological motion (Miura et al. 2018). Corticosteroid action terminates imprinting learning (Weiss et al. 1977; Yamaguchi et al. 2012), which is also consistent with their role in insecure attachment organisation in humans (Lemche et al. 2005). The onset of myelination of the visual system stabilises these circuits and inhibits further imprinting learning (Yamaguchi et al. 2012). However, Dio2 in telencephalon including subpallial areas (Takemura et al. 2018) was found capable to reactivate imprinting learning towards stimuli with biomotion preference, and thus may be also critical for early social bonding.

Further to T_4_, as was shown recently (Batista et al. 2018), is the activation of the Akt/mTOR pathway in IMM and MNM essential for restoring imprinted memories *beyond* the experience-expectant sensitive period. The mechanistic target of rapamycin complex 1 (mTORC1) is a protein complex involved in sensing nutrients and regulating protein synthesis triggered by signalling of insulin and its growth factors. Experience-dependent neuronal plasticity in outgrowth of dendritic spines is triggered by mTORC1, and it was shown that mTORC1 determines pre-synaptic spine morphology (Batista et al. 2018) in neuronal circuitry formed upon visual and auditory imprinting.

## Prewired preferences for face patterns and biological motion

Electrophysiological frequency tagged responses and source modelling in human infants support the long-ascertained (for review see Lemche (1997)) existence of “prewired” face-pattern templates active postnatally, which are consistent with a subcortical fast-tract face-recognition route involving pulvinar, superior colliculus and amygdala (Johnson et al. 2015), next to slower cortical modules fusiform face area (FFA), praecuneus (Buiatti et al. 2019) and other face-selective neuron bearing cortical regions (Buiatti et al. 2019; Lemche 2003). These routes are consistent with Horn’s two-process theory of imprinting (Horn 2004; Johnson et al. 2015), with its conjecture of facial cue predisposition and memory engramme formation by social learning.

Newborns at familial risk for autistic spectrum tested for face-like patterns and biological motion did not exhibit the inborn face preference or predisposition, but, in contrast, showed preferences for non-biological motion and inverted faces, discriminative at ROC 0.85 (Di Giorgio et al. 2016). Neonatal domestic chicken also show inborn predispositions for face-like patterns and self-propelled biological motion (Di Giorgio et al. 2017; Lorenzi et al. 2019). Thus, there is a perceptual parallelism between chicks and human infants in face-like and biomotion priors for imprinting, also beyond the sensitive period.

## Historical roots of human attachment theory

The translation of ethological findings into psychology are fruit of the scientific efforts of psychoanalyst John Bowlby (1907–1990), who had personally experienced early nanny care in a nobility family environment. He sought to provide an empirical basis for mental “object relation” experiences during psychotherapy. Bowlby (1960) traced human separation anxieties and compared it with separation distress behaviours in animal infants. Later work expanded towards secure base behaviours, grief after losses, and internal working models of attachment relationships. His collaborator psychologist Mary Ainsworth conducted field observations amongst the Ganda ethnicity, and so informed thereafter devised the strange situation experimental paradigm (Ainsworth and Bell 1970), which enabled probing human attachment quality by separation and an intruding stranger prior to caregiver reunion.

## Established developmental antecedents of human attachment security or insecurity

Since the description of the strange situation procedure, with its discrimination in secure vs. insecure attachment relations (later: four attachment classifications), prospective studies tried to identify the causes for attachment security. Recurrent facial and vocal synchronies (Isabella and Belsky 1991) inducing shared positive emotional states in the postnatal 3-month window are the hitherto best supported attachment predictors. Rejection, aversive, and subtle negative emotion signals are assumed to generate separation distress in infants conducive to later insecurity (Lemche et al. 2005). Maternal sensitivity, a stance of empathic accompaniment of infant behaviours with similar vocal and gestural contours in “consistent contingency”, is the best-replicated prerequisite of attachment security: meta-analyses (Atkinson et al. 2000) average their correlations at around *r* = 0.27.

## Established cognitive-representational nature of attachment patterns

Bowlby assumed cognitive attachment representations underlying behavioural pattering. Empirically had the 7-month weariness towards strangers as different from caregivers been accepted as an indicator of a mental representation of the caretaker; a similar mechanism is the visual recognition of kin in rodents (Ferguson et al. 2001), also known in sheep (Kendrick et al. 1998). Both childhood (Inge Bretherton) and adult (Mary Main) interview methods established a narrative dimension of attachment representations. Longitudinal studies confirmed that verbal indices of mental person representations (Lemche 2003) develop in dependence of attachment classifications further to language development (Lemche et al. 2013). Life-span relative stability of attachment representation into adulthood has been found modifiable by reflection of attachment memories.

Very well established are culture-specific distributions of attachment classifications and suggest possible varying emotional caretaking habits amongst different human populations. Maternal attachment representations predict infantile attachment patterns at a probability of 80%, thus further supporting a cognitive-representational nature of attachment relations.

## Unsupported causes: temperament, personality, and Mendelian genetics

Vitale and co-authors (2019) state that feline attachment patterns are possibly based on temperament. This long-standing assumption has now been largely refuted by meta-analysis (Groh et al. 2017) in humans. There is also no evidence for an association of attachment classification with personality constructs (e.g. Big Five); however, marginal association was found for questionnaire-based adult attachment styles with the NEO-FFI.

These authors (Vitale et al. 2019) also state that attachment patterns in pets are possibly based on hereditary traits. To date, there is no strong support for single-point genomic (*OXTR* for attachment classification; *OXTR*, *CD38* for sensitivity; *DRD4*, *HTR2A*, *SCL6A4*, *COMT* for attachment insecurity/disorganisation) effects, which are therefore disputed. Implications of dopamine, serotonin and catecholamines point to general stress diathesis in attachment formation under risk. Possible epigenetic mechanisms, as suggested by findings in ELS studies (Lemche 2017), would perhaps better match human-raised pet results:

It was shown in rodents (and in humans) that the hypothalamic oxytocin receptor promoter is subject to epigenetic modification by DNA methylation, which regulates *Oxtr* transcription (Mamrut et al. 2013); *Oxtr* expression was found highest in medial amygdala and olfactory structures in rodents (Harony-Nicolas et al. 2014), and reciprocally associated with differential methylation sites in the *Oxtr* promoter region. Initial synchronic and diachronic evidence supports epigenetic modification of the *OXTR* (promoter, enhancer, intron 1 CpG island methylation) (Ein-Dor et al. 2018; Gouin et al. 2017; Unternaehrer et al. 2015) in humans. Activation of *OXTR* triggers Gq, a heterotrimeric G-protein subunit, which is part of the MAPK signalling pathway (Feldman et al. 2016).

The ectoenzyme CD38/adenosine diphosphate-ribosyl cyclase is a transmembrane receptor confined to the brain, whose activation triggers downstream intracellular calcium signalling pathways, critical for oxytocin release (Jin et al. 2007), and covaried with T_3_ (Gao et al. 2018). In human early developmental studies (reviewed in Feldman et al. (2016)), *CD38* point mutations were associated with parental touch, interactional synchronies and parent-infant reciprocity (predictive of secure attachment).

## Cerebral regulation of attachment security

**Fig. 1:**
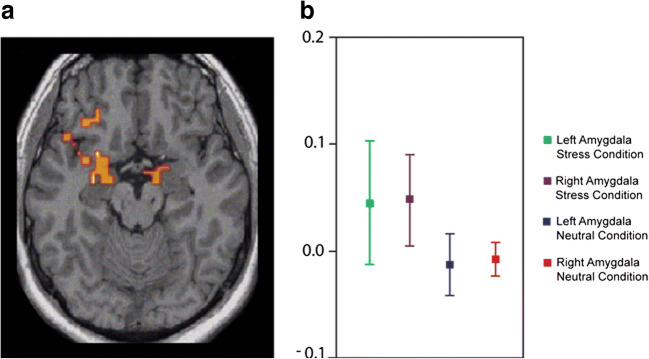
**a** Bilateral amygdala activation in an experimental paradigm inducing separation distress (stress condition). **b** BOLD signal levels in the left and right amygdala in stress and neutral experimental conditions, the neutral condition with relative amygdalar deactivation consistent with attachment security (reproduced with permission from Lemche et al. (2005))

Of the number of neuroimaging studies on attachment, few were able to experimentally probe filial separation distress and describe cerebral mechanisms of its activation. By combination of fMRI and peripherally measured sympathetic arousal in parallel (Lemche et al. 2005), there was support for human homologue structures engaged as in avian experimental results (the arcopallium/archistriatum/amygdala) (Fig. 1). There is conclusive evidence that the attachment security-insecurity continuum is being regulated by bilateral amygdala/globus pallidus, with greater sympathetic outflow indicating insecurity-related stress proneness. Amygdalar deactivation, ventral striatal and ventral tegmental area activation (VTA, origin of the dopaminergic neurones in the mesocorticolimbic system) are currently envisaged as subserving secure base experience (Long et al. 2020). Corresponding brain regions for maternal bonding to infants were ascertained in amygdala, ventral striatum and pallidum (Atzil et al. 2017).

## Early life stress research and metabolic consequences

Rodent early life stress (ELS) research has established maternal separation as one central experimental paradigm. ELS research is supportive of cerebral functional and structural alterations, along with long-term metabolic sequelae (Lemche 2017), following early maternal separation. These, in particular, include abnormalities in amygdalar and hippocampal volumes and activation, as also found in human brain activation studies (Lemche et al. 2005). The results are consistent with human developmental brain studies documenting respective abnormalities following early traumatic experiences or abuses. The autonomic imbalance following chronic separation distress, triggering HPA hyperreactivity, is a major source of metabolic alterations towards biasing towards later insulin resistance (Lemche 2017).

## Attachment in animal research: species specificity

A hitherto largely unanswered issue is the species specificity of attachment patterns exhibited by mammals towards human caretakers. The published pet studies suggest that both feline and canine infants and adults exhibit similar general attachment behaviours in the secure-base tests applied. With regard to weariness towards strangers, the question arises: is it specific to human and non-human primates (e.g. chimpanzee, macaques) (Bauman et al. 2004; Miller et al. 1990)? In the studies published, this was also confirmed for dogs/wolves and cats (Vitale et al. 2019). It is not known if this is also present in sheep, goats (Kendrick et al. 1998; Neumann 2008) or *Octodon degus* (Zehle et al. 2007) also investigated. The caretaker preference would be an indicator for exclusive bonds postulated by attachment theory.

## Brief summary of new questions arising from research on companion animals

Given the importance of human attachment figures in human-raised pet studies, resulting questions are: Are pet infants making the same experiences as human infants with their human caregivers? Did cubs or puppies not imprint to their physical mothers? Are mechanisms of maintenance and stability introduced ontogenetically? How was the experimental control of imprinting-analogue experiences in the animal subjects (e.g. kitten and puppies are known susceptible to later tolerating human contact only up to 12 postnatal weeks)? Do pets show indications of a development of mental person representations in terms of social cognition? Is feeding the causation of human-like attachment patterning? Is sensitive parenting on the owner side with emotional facial and vocal stimulation the cause of attachment-like bonding behaviours towards the human caregiver? Are epigenetic modifications the key to attachment stability in animals and humans?

## Conclusion and directions for future research

The reviewed findings from the fields of filial imprinting and early attachment indicated that, despite common starting points, the fields have moved on in separated trajectories. It has become apparent that few research has hitherto attempted to integrate the hypotheses and findings of the other field. Whereas experiments in filial imprinting have aimed at elucidating neuronal plasticity in cognitive and memory domains, a great deal of work on human or rodent attachment was based on the oxytocin hypothesis (Insel 1992), the dopamine hypothesis (Strathearn 2011) or the opioid hypothesis of attachment (Herman and Panksepp 1981; Machin and Dunbar 2011). These hypotheses have been investigated in avian mating (Numan 2015), but systematic probing in filial imprinting research has only yet begun (Loveland et al. 2019), despite the circumstance that e.g. avian species have mesotocin/isotocin/arginine vasotocin systems homologous to the prosocial nonapeptides in mammals.

The demonstration that thyroid hormone T_3_ regulates the sensitive period for imprinting learning and memory consolidation opens a new route for such integration. It is long established that T_3_ activates the mammalian *OXT* gene promoter independently of oestrogen (Adan et al. 1992), triggering hypothalamic-neurohypophyseal oxytocin release (Adan et al. 1992; Ciosek and Drobnik 2004). Moreover, it has previously been described that TRH-secreting neurones in the hypothalamus converge with dopaminergic neurones in regulation of thyroid hormones and brain growth (Reymond and Lemarchand-Béraud 1990). Although DA neurones lack TRalpha1 receptors, it has been demonstrated that T_3_ exerts neuroprotective and proliferative effects on DA neurones and on oligodendrocytes (Lee et al. 2019), thus promoting myelination. TRalpha1 is the prevailing TR isoform in the brain and present in early embryonic development (Schroeder and Privalsky 2014). Peripheral T_4_ transgresses the BBB through endothelial cells with transporter OATP1C1, and transporter MTC8 provides entry of T_3_ into neurones and oligodendrocytes after Dio2 conversion within tanycytes (Schroeder and Privalsky 2014).

The magnocellular oxytocin releasing neurones and the parvicellular TRH-releasing neurones (Clasadonte and Prevot 2018) in the paraventricular nucleus are embedded in the endfeet of radial glia-like tanycytes. Furthermore, tanycytes are the major Dio2 expressing cells, and also the T_4_ to T_3_ conversion exerted by Dio2 occurs in the tanycytes of the median eminence (Clasadonte and Prevot 2018). These anatomical enmeshments bear implications for various metabolic processes and inflammatory states (Gao et al. 2018) described in ELS studies.

Hence, it is recommended that attachment research should integrate recent advancements in filial imprinting (regarding thyroid hormones) in its hypotheses and experimentation, whilst research in filial imprinting should make use of neuroendocrine findings in attachment research. This integration would also strengthen the study of metabolic consequences of insecure attachment and early life stress.
